# Body stalk anomaly presenting as an emergency in Ethiopia: a case report

**DOI:** 10.1186/s13256-020-02509-3

**Published:** 2020-10-29

**Authors:** Tafese Dejene Jidha, Tekle Wakjira, Tigist Mekonnen

**Affiliations:** Department of Obstetrics & Gynecology, Jimma University Medical Center, Jimma, Ethiopia

**Keywords:** Body stalk anomaly, Primigravida, Third-trimester pregnancy, Bradycardia

## Abstract

**Background:**

Body stalk anomaly is a generally lethal malformation of the thorax and/or abdomen. It is often associated with limb defects. The intrathoracic and abdominal organs lie outside the abdominal cavity. These are contained within a sac composed of amnioperitoneal membrane attached directly to the placenta. The umbilical cord may be totally absent or extremely shortened. Severe kyphoscoliosis is often present. This case is reported to highlight this rare malformation and its clinical presentation.

**Case presentation:**

We present a case of a 27-year-old primigravida Oromo woman who had been amenorrheic for 9 months. She presented with an urge to bear down of 10 hours and passage of liquor of the same duration. The patient was referred from a district primary hospital with a diagnosis of preterm labor and hand prolapse. A lower uterine segment cesarean section was performed at Jimma University Medical Center for an indication of active first stage of labor with nonreassuring fetal heart rate pattern (fetal bradycardia) and hand prolapse to effect an anomalous fetus that had only a rudimentary right lower extremity and liver and intestine found outside the abdominal cavity contained within a sac composed of transparent membrane attached directly to the placenta. The umbilical cord was very short, measuring about 7 cm. The fetus had severe scoliosis. It also had a heartbeat upon extraction, which stopped after 5 minutes of delivery. The placenta and fetal body parts together weighed 2400 g.

**Conclusion:**

Termination of pregnancy is usually offered because this abnormality is generally considered lethal. If the pregnancy is continued undetected as in our patient’s case, vaginal delivery is recommended, given the highly lethal nature of this anomaly. Good prenatal screening and counseling are recommended for early detection and management.

## Background

The disease known as body stalk anomaly (BSA), limb body wall complex (LBWC), or body stalk complex is a very rare anomaly (1 in 14,000 to 42,000 pregnancies; 1 in 7500 fetuses from 10 to 14 weeks of gestation). This medical condition is characterized by a complex anomaly of the anterior abdominal wall, severe kyphoscoliosis, rudimentary umbilical cord, and anatomical defects of the pelvis and lower limbs [1–3]. LBWC has no sex or familial predilection or known recurrence risk. Karyotype study has been reported to be normal in all cases of LBWC. The pathogenesis of this variable spectrum of anomalies is not well understood, but it is usually incompatible with life, progressing to miscarriage or a stillborn fetus. We report a case of BSA presenting in the third trimester on an emergency basis for which cesarean section (C/S) was performed for a nonviable fetus. Such a condition is common in developing countries where ultrasound and prenatal screening are rarely available.

## Case presentation

A 27-year-old primigravida Oromo woman who did not remember her last normal menstrual period, but who claimed amenorrhea of 9 months’ duration, presented to Jimma University Medical Center. She received antenatal care (ANC) follow-up at her local health center and was referred from the district hospital with a diagnosis of preterm labor and hand prolapse. She presented with an urge to bear down of 10 hours’ duration and passage of liquor of the same duration. She did not undergo ultrasound scanning during ANC follow-up. Her personal, familial, medical, and obstetric histories were unremarkable. The pregnancy was planned, wanted, and supported.

Upon arrival, she was in labor pain, and her vital signs were blood pressure 110/70 mmHg, pulse rate 96 beats per minute, respiratory rate 22 breaths per minute, and body temperature 37 °C. A pertinent finding was detected on the abdomen: a 28-week-sized gravid uterus, with the fundus occupied with a round, hard mass that was the head. Fetal heartbeat (FHB) was 92 to 98 beats/minute, and uterine contractions were 3 in 10 minutes lasting for 40 to 45 seconds. The patient’s pelvic examination revealed a cervix of 6-cm dilation, membrane ruptured and clear, station high, and left hand prolapsed and in the vaginal canal. Emergency obstetric ultrasound was performed, and the finding was a single intrauterine pregnancy, positive FHB ranging from 92 to 98 beats/minute, fundal anterior placenta, and head occupying the fundus.

**Fig. 1:**
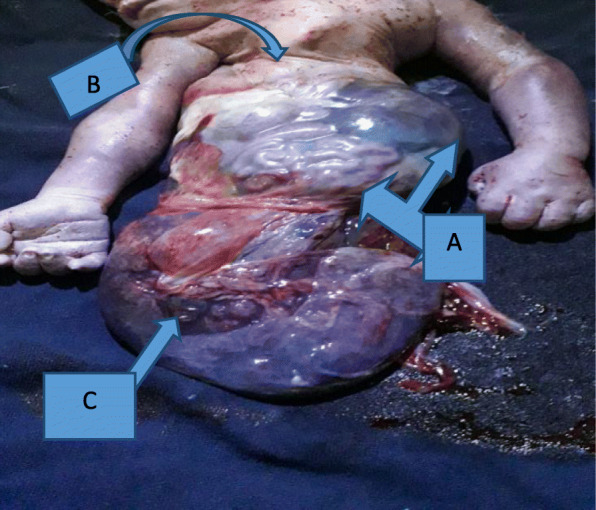
Neonate with body stalk malformation. **a** Liver and intestine outside abdominal cavity covered with a transparent membrane. **b** Malrotation at chest level. **c** Maternal surface of placenta shown

**Fig. 2:**
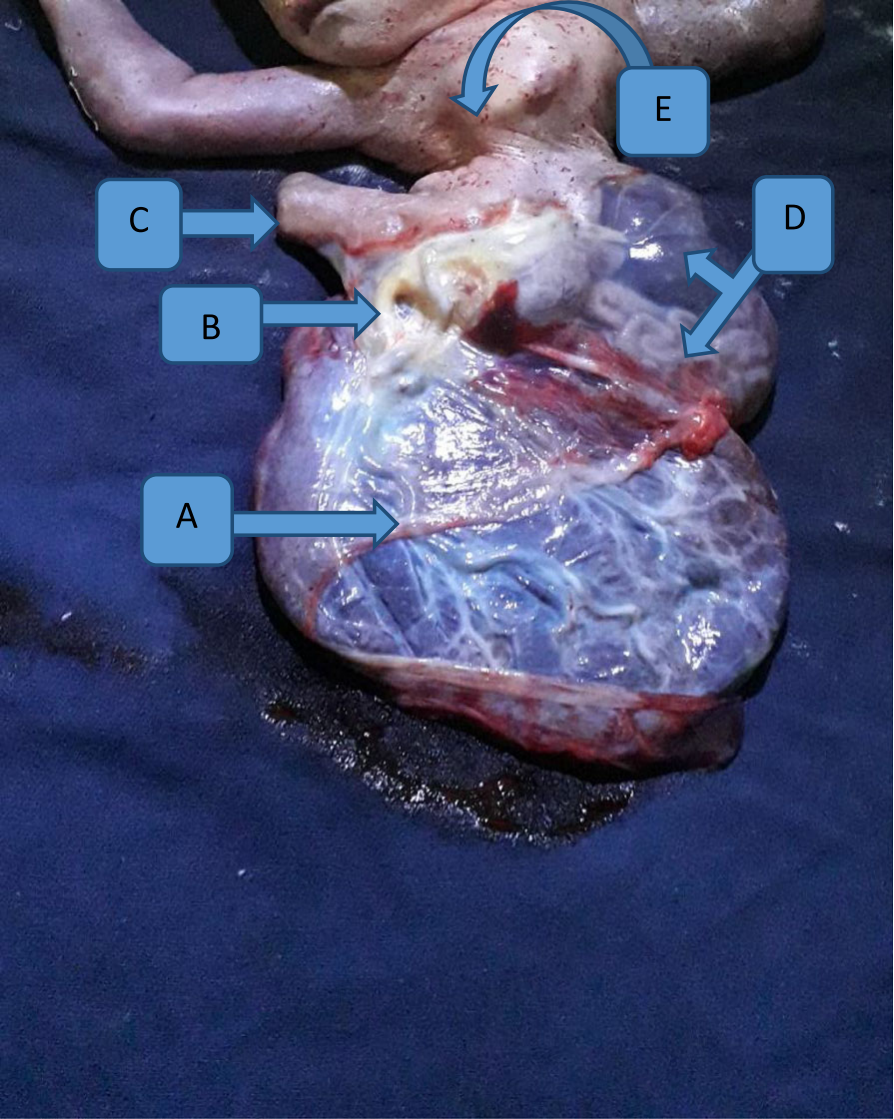
Neonate with body stalk malformation. **a** Fetal surface of placenta. **b** Short cord measuring about 7 cm. **c** Right side rudimentary lower extremity. **d** Liver and intestine covered with transparent membrane. **e** Malrotated chest

With the impression of active first stage of labor with hand prolapse and nonreassuring fetal heart rate pattern (NRFHRP [persistent fetal bradycardia]), the patient was investigated with blood group and Rh (O+), hematocrit (37%). Then, she was prepared and taken to the operating theater for emergency C/S within 20 minutes of arrival. Transverse lower uterine segment C/S was performed with the patient under general anesthesia to effect the delivery of an anomalous fetus with Apgar scores of 3, 3, and 0 in the first, fifth, and tenth minutes, respectively. The fetus had only a rudimentary right lower extremity. The liver and intestine lied outside the abdominal cavity and were contained within a sac composed of transparent membrane attached directly to the placenta. The umbilical cord was very short, about 7 cm. The fetus also had severe scoliosis. The placenta and fetal body parts together weighed 2400 g (Figs. 1 and 2). The newborn lived only for 5 minutes after delivery, and an autopsy was not performed. The patient had no complications during or after surgery, and her postoperative hematocrit was 34%. She was discharged on her third postoperative day. She was counseled and provided an etonogestrel implant. She was given a C/S certificate that she can try labor with subsequent pregnancies.

## Discussion

This case report describes an extremely rare presentation of BSA diagnosed after delivery by C/S on emergency base after 9 months of amenorrhea. “Body stalk anomaly” is a term used to describe a pattern of severe defects that in most of the reported cases prove to be incompatible with life. This condition should be suspected when a large abdominal defect as well as abnormalities in the axial skeleton such as kyphosis or scoliosis are observed and a short or absent umbilical cord is found. Other pathologies that affect the anterior abdominal wall are omphalocele, gastroschisis, and pentalogy of Cantrell.

Gastroschisis is generally a full-thickness right paraumbilical defect in the anterior abdominal wall (2–5 cm) through which the intestinal contents freely protrude. No covering membrane is present, and cord inserts are seen on the abdominal wall, with only loops of bowel herniated. Pentalogy of Cantrell is a cluster of defects first described by Cantrell in 1958 in five neonates. It is characterized by an upper midline omphalocele, anterior diaphragmatic hernia, ectopia cordis, sternal cleft, and intracardiac defects. Omphalocele is a midline defect of the abdominal wall; generally, the defect size is 2–15 cm, and herniated content is covered with membrane with umbilical cord inserts on top of the membrane [4, 5].

In our patient’s case, the liver and intestine lied outside the abdominal cavity and were contained within a sac composed of a transparent membrane attached directly to the placenta. The umbilical cord was very short, about 7 cm. The fetus had also severe scoliosis and a rudimentary right lower extremity, which made the diagnosis of BSA.

The pathogenesis of BSA is not clear. However, there are a few theories to explain this defect. The first theory is early amniotic rupture theory (exogenic theory). The theory claims that multiple amniotic bands interrupt embryogenesis and make the fetus to lie outside the amniotic cavity in BSA [6]. Recently, different studies have failed to demonstrate any evidence of amniotic rupture and fibrotic bands in the presence of BSA and therefore challenged the validity of this theory [7]. The second theory is vascular disruption (endogenous theory). Here, events negatively influence normal embryonic blood supply, interrupting normal morphogenesis [8, 9].

Some studies showed the association of BSA and maternal cocaine abuse [10, 11]. Impaired placental perfusion during critical periods of development due to the vasoconstrictive effect of the drug may be the underlying mechanism in these instances. We did not perform drug testing in our patient, but she had no history of drug abuse or use of vasoactive medication. The most commonly accepted theory is Streeter’s 1930 theory of abnormal embryonic folding [12], modified in 1989 by Hartwig [13].

During the fifth week of gestation, the flat trilaminar embryo is transformed into a cylindrical fetus by a parallel set of four contiguous body folds (cephalic, caudal, and both lateral folds). Maldevelopment of each of the four folds results in a distinct constellation of anomalies [14]. Abnormal cephalic folding may produce a form of pentalogy of Cantrell. Even lateral fold defects may result in omphalocele. However, aberrant caudal folding may create any or all of the abnormalities of cloacal exstrophy. BSA is supposed to be due to faulty folding in all three axes with persistence of the extraembryonic celomic cavity. Depending on the degree of aberrant development of each fold, various malformations are associated with BSA.

Van Allen *et al.* set forth the diagnostic criteria for BSA in 1987. Two of the following three anomalies must be present to establish a positive diagnosis [8, 15]:

- Exencephaly/encephalocele with facial clefts
- Thoracoschisis and abdominoschisis (midline defect)
- Limb defect (for example, clubfoot, polydactyly, oligodactyly, syndactyly, brachydactyly, amelia)

In 1993, Russo *et al.* postulated the presence of two clearly distinguishable phenotypes [16, 17] as a consequence of different pathogenetic mechanisms [17]. The first phenotype presents craniofacial defects, amniotic bands, and/or adhesions. Here, the pathogenic mechanism proposed is early vascular disruption. The second is attributable to an intrinsic embryonal maldevelopment and shows no craniofacial defects but urogenital anomalies, anal atresia, and abdominal placental attachment together with persistence of the extraembryonic celomic cavity. In our patient’s case, no craniofacial defects were present, but we found persistence of the extraembryonic celom and abdominal placental attachment. Therefore, our patient’s case belongs to the second phenotype because of embryonic maldevelopment.

The disease is invariably fatal, and BSA needs an early antenatal diagnosis and termination of pregnancy. BSA is most frequently diagnosed during the second trimester of pregnancy with ultrasound. Case reports in the third trimester are rare. Our patient presented in the third trimester. However, antepartum and intrapartum diagnosis of BSA was missed. The reason was that she had no ultrasound anatomic screening during antenatal care follow-up, and emergent presentation of our patient to our hospital in labor with NRFHRP (fetal bradycardia) necessitated immediate delivery. As a result, we could not get a detailed sonographic anatomic survey. The diagnosis of BSA was made postnatally after delivered by emergency C/S. A comparison of this case with other case reports published previously is presented in Table 1. If our patient’s case had been diagnosed antenatally, she should have been allowed to deliver vaginally because of the incompatibility with life.

**Table 1:** Review of the literature on body stalk malformation

Reference	Description of study	Type of malformation	Outcome
Singh *et al*., 2017 [5]	Case report	Large omphalocele with herniation of the small intestine and liver covered by the amniotic membrane and attached to the placenta Very short umbilical cord	Abortion at 15 weeks
Kocherla *et al*., 2015 [15]	Case report	Large omphalocele with herniation of the small intestine and liver covered by amniotic membrane Lower limbs deformity with bilateral talipes Anencephaly with spina bifida Karyotype 46,XX	Abortion at 14 weeks
Plakkal *et al*., 2008 [16]	Case report	Very short umbilical cord Amelia of the left upper limb Large defect in the body wall involving the thorax and the abdomen with evisceration of the lungs, stomach, liver, spleen, and intestines The sternum, the anterior costal parts of the ribs on the left side, and the diaphragm were absent. Scoliosis of the spine	Stillborn at 36 weeks
Luiz *et al*., 2016 [18]	Case report	Bulky ruptured omphalocele Pelvic asymmetry Atrophy of the lower limbs with clubfoot	Delivered by cesarean section at 36 weeks of gestation Survived for 84 days
Maria *et al*., 2016 [19]	Case report	Severe kyphoscoliotic spine Large anterior abdomen wall with liver, stomach, small and large bowels, right kidney, cardiac apex, and two right lung lobes outside the body with no membrane surrounding them Congenital diaphragmatic hernia with the ascent of the left kidney in the thoracic cavity, along with hypoplastic lungs, with three lobes on the right lung and four lobes in the left one Internal hydrocephalus in the brain Short umbilical cord	Abortion at 23 weeks
Adelita and Melissa, 2015 [20]	Case report	Extrusion of the intra-abdominal and thoracic contents Syndactyly of the third and fourth fingers of the right hand 8-cm umbilical cord with two arteries and one vein Permeable anus	Delivery by cesarean section at 27 weeks of gestation Died immediately
Our patient	Case report	Fetus had only rudimentary right lower extremity Liver and intestine lie outside the abdominal cavity covered by a transparent membrane Very short umbilical (7 cm) cord Severe scoliosis	Delivered by cesarean section Died immediately after delivery

### Patient’s perspective

I first found that I was pregnant when I missed my first period and went to Asendabo Health Center. The center did a urine test and told me I was pregnant. I remember how happy my husband and I were because I became pregnant after 1½ years of our marriage. The physician advised me to start follow-up for my baby, and I returned to the health center at my fourth month of amenorrhea. The physician took my blood pressure; examined my chest and abdomen; and ordered blood, stool, and urine tests. He told me everything was okay, gave me one injection over my left upper arm, and appointed me to come after a month. He also told me that the center had no ultrasound, so he advised me to go to Jimma town and have scanning for my baby. However, I didn’t go, because I didn’t think it was important.

I went to the health center for my second visit at my sixth month of amenorrhea, after 2 months of my first visit. I didn’t go at fifth month, because I had no problem, and that was why I went after 2 months. During that time, the physician again took my blood pressure, examined my chest and abdomen, then he told me everything was okay. The physician also put one circular tube over my abdomen and told me he heard my baby’s heartbeat. He gave me an injection over my left upper arm and red tablets to take one tablet daily for 3 months. He appointed me to come after a month.

I went for my third visit after a month. He took my pressure, examined my chest and abdomen. He said that he heard my baby’s heartbeat with a circular plastic tube. He told me everything was good and advised me to come after 2 weeks. I didn’t return after 2 weeks, because I had no problem, and also my baby was moving well in my abdomen.

In the early morning of 29 September 2011, according to Ethiopian calendar (which corresponded to June 6, 2019 Gregorian calendar), I planned to go to market and started walking. On my way, I bore down, and clear fluid gushed out of my vagina. I immediately returned home and went to the health center. In the center, the physician examined me and told me that one hand of my baby was coming ahead of head. He urged me to go to Nada Hospital, which is about 10 km from my home. At the hospital, the physician examined me and gave me a referral letter so I could go to Jimma University Hospital for operation because Nada Hospital had no electricity by the time.

At Jimma University Hospital, physicians examined me and saw my baby with ultrasound. They told me that one hand of my baby was coming out ahead of my baby’s head. They also told me that my baby’s heartbeat was so slow that if I did not undergo an operation, my baby might die. So, they immediately signed me the consent, and took me to the operation room. Then, I felt that would die. Anesthetist came and told me he would give me a drug that would make me sleep during the operation. He informed me I would not feel any pain during the operation.

When I woke up after the operation, I felt fine, though I had minimal pain at the surgical wound site. I asked a female doctor who was following me up to show me my baby. She told me that my baby had a severe congenital anomaly, and it couldn’t survive and died immediately after delivery. Then, my husband and I started crying. I was in deep grief, and my tears ran out of my eyes when I saw other mothers carrying their babies, feeding breast. The doctor explained to me everything about the condition of my baby. It worried me that what I should do in order not to have such problem in case if I become pregnant again. However, she told me it will not recur and informed me to have an early ultrasound with next pregnancy to check the baby for abnormality.

When she told me about the importance of ultrasound for early detection of abnormality, I remembered what the physician at the health center who advised me to have ultrasound for checking of my baby, but I didn’t do, because I didn’t consider it was that much important, but I regret. If I had had an ultrasound, I wouldn’t have carried a nonsurviving fetus, and I wouldn’t have been operated. Now, I understood the importance of physicians’ advice and the importance of ultrasound. I will have an ultrasound if I become pregnant, and even I will advise pregnant ladies at my village to have ultrasound during follow-up.

## Conclusion

Termination of pregnancy is usually offered because the abnormality is generally lethal. If the pregnancy is continued undetected, as in our patient’s case, vaginal delivery is recommended only if there is no contraindication for vaginal delivery. Yet, because of the lethal nature of the anomaly, the patient should be extensively counseled on the prognosis of the fetus. The family must be reassured that there is no risk for recurrence. Good prenatal screening and counseling are recommended for early detection and management.

## Data Availability

Data sharing is not applicable to this article as no datasets were generated or analyzed during the current study.
